# Effectiveness of game-based virtual reality phone application and online education on knowledge, attitude and compliance of standard precautions among nursing students

**DOI:** 10.1371/journal.pone.0275130

**Published:** 2022-11-03

**Authors:** Khaild AL-Mugheed, Nurhan Bayraktar, Mohammad Al-Bsheish, Adi AlSyouf, Badr K. Aldhmadi, Mu’taman Jarrar, Moath Alkhazali

**Affiliations:** 1 Surgical Nursing Department, Faculty of Nursing, Near East University, Nicosia, Cyprus; 2 Nursing Department Kizilcasar Mahallesi, Atılım University School of Health Sciences, Golbasi, Ankara, Turkey; 3 Healthcare Management Department, Batterjee Medical College, Jeddah, Saudi Arabia; 4 Department of Managing Health Services and Hospitals, Faculty of Business Rabigh, College of Business (COB), King Abdulaziz University, Jeddah, Saudi Arabia; 5 Department of Health Management, College of Public Health and Health Informatics, University of Ha’il, Ha’il, Saudi Arabia; 6 Vice Deanship for Quality and Development, College of Medicine, Imam Abdulrahman Bin Faisal University, Dammam, Saudi Arabia; 7 Medical Education Department, King Fahd Hospital of the University, Al-Khobar, Saudi Arabia; 8 Surgical Nursing Department, Faculty of Nursing, Nicosia, European University of Lefke, Lefke, Cyprus; Central China Normal University, CHINA

## Abstract

Game-based virtual reality phone applications can create a realistic environment to prepare for clinical applications and improve students’ knowledge of and compliance with standard precautions. An experimental study was performed among 126 nursing students’ from the third and fourth nursing levels to determine the effect of online education and game-based virtual reality phone applications related to standard precautions. Students were divided randomly into two groups; the experimental group used online education and game-based virtual reality phone applications, while the control group used traditional education. The study was performed between July and August 2019 to prevent clashes with lectures and midterm and final examinations. A tool package including knowledge, attitude, and compliance with standard precautions was used in pre-and post-tests among nursing students. The results showed that the knowledge of, attitudes towards, and compliance with standard precautions differed between the two groups. The performance of the experimental group of nursing students significantly improved with online instruction and game-based virtual reality phone applications. This study demonstrated the effectiveness of online education and game-based virtual reality phone application among nursing students.

## Introduction

A Hospital-Acquired infection (HAI) is an infection created in a hospital setting that was neither incubating at the time of admission nor represented two days or more after admission [1]. The World Health Organization emphasizes that HAIs have become a crucial and universal issue for patient safety, impacting hundreds of millions globally [2]. HAIs are a concern in developing nations, impacting up to 25% of hospitalized patients [3]. HAI contributes to prolonged hospitalization, lengthened patient care, increased care expenses, and increased suffering of patients and their families due to HAI complications [4, 5].

The World Health Organization considers standard precautions the first line of defense for preventing the spread of infections [2]. Standard precautions are principles and practices applied to protect a healthcare professional’s team and patients and reduce the risk of transmission from pathogens [1]. These precautions include hand washing, hand rub, gowns, gloves, masks, goggles, and face shields [1]. Studies of low- and middle-income countries indicated that nursing students’ knowledge, attitude, and compliance with standard precautions were low and inadequate [6, 7]. Al-Rawajfah and Tubaishat (2015) conducted a study that evaluated knowledge and practice of standard precautions among nursing students in Jordan; students had overall satisfactory knowledge, with a mean of 13.8 out of 18 [6]. In Darawad and Al-Hussami’s study, students showed inadequate knowledge of infection control precautions. In a multi-university study in Saudi Arabia, nursing students reported moderate compliance regarding standard precautions during their clinical practice [7]. These results may be attributed to dissatisfaction and lack of infection control course content in nursing schools [8], weak clinical experience, or lack protective supplies [9]. During clinical training, these might impact students’ compliance and performance regarding standard precautions skills and may become a cross-infection source [9]. In this regard, prevention and education are significant aspects of nursing.

The educational process of health professions like nursing is unique because a student must not just understand concepts but be able to use practices in real-life situations, which are desired competencies for graduation [10]. Nursing lecturers face several challenges in modern teaching methods in transitioning a student from an inexperienced to an experienced practitioner [11, 12]. For this reason, using an innovative approach, like virtual reality, is essential.

In recent years, online education and new technologies have increased in nursing education. Online education has been supported for its capacity to de-centralize the teaching process and evolve from conventional learning to a student-centered approach [13, 14]. This approach allows students to use different technologies to help them understand educational lectures prior to the class and then to apply this knowledge based on instructors supervision [15]. Online education allows faculty lecturer to engage large groups of students simultaneously with synchronous and asynchronous learning options [16]. During online classes, students can utilize time via problem solving, group discussions, and active learning activities [17]. Several studies indicated that online education is an innovative method, helps with knowledge retention, and is a more exciting way to acquire knowledge about standard precautions than other tactics [18, 19].

Virtual reality (VR) is an artificial computer-created setting examined by sensory stimuli and is a social constructivism approach to learning [20]. It is a powerful education method that engages students in active participation, problem-solving and interactive teaching [21]. VR is a three-dimensional computer-based simulation that reinforces pragmatic student education in a clinical setting. It provides students the feeling of being present engaging their sensory organs [22, 23]. A virtual environment facilitates and boosts the education of many students at once and at locations without instructor engagement during undertaking learning or practice [24]. The literature indicates that virtual reality improves nursing students’ knowledge and skills outcomes compared to traditional approaches [25, 26]. A review identifying the impacts of integrating virtual reality in nursing education programs found increased enjoyment and engagement in learning, a willingness to learn without feeling bored, and enhanced competence and confidence among students [23].

Along these lines, a game-based virtual reality phone application is a gamification technology that offers students opportunities to experience real-life perspectives and touch things not actually there [25]. Using a game-based virtual reality phone application, a teacher creates a game scenario and content and uploads it to a phone application. Then, pre-class learning based on the game is conducted, the application is downloaded, and the game is played [11, 27]. A game-based virtual reality phone application capitalizes on features that dramatize a situation, make participating fun, and utilize outcomes for learning [25]. In this method, the content of games is drawn from realist-life situations and shifted to computer situations on mobile phones, tablets, and iPad devices. This method allows students access to information at any time and place and assists students in gaining individual theoretical and practical time to achieve skill proficiency [11, 25]. Several advantages of game-based virtual have been noticed. These include improved skills and knowledge, self-efficacy, psychomotor skills, and self-learning [25, 27]. Therefore, it enhances student satisfaction, retention in the learning process, and easy access to information [26].

Using two educational approaches online instruction and virtual reality have significantly improved knowledge acquisition and satisfaction among undergraduate nursing students [19, 28–30]. Furthermore, it has great potential in providing experiential, repeatable learning opportunities for students to learn collaboratively [31, 32]. The learning outcomes involved not only pedagogical achievements (e.g. knowledge), but also enjoyment, satisfaction, and clinical experience [33]. Clinical experience offers sense to the learning process, allowing for authentic and genuine learning to occur [34]. Existing evidence suggests that positive clinical experiences increase feelings of enjoyment and satisfaction, which translate into applying gained knowledge to clinical practice [35]. Further studies are needed to increase evidence regarding the effectiveness of innovative educational methods to improve the quality of nursing education.

### Objectives

The study’s main objective was to assess the effectiveness of online instruction and game-based virtual reality phone applications compared with traditional education related to knowledge, attitudes, and compliance with standard precautions among nursing students.

### Research hypotheses

H1. Nursing students who complete online instruction will have higher knowledge toward standard precautions than the traditional educational group.

H2. Nursing students who complete online instruction will have higher attitudes toward standard precautions than the traditional educational group.

H3. Nursing students who complete a game-based VR phone application will exhibit higher compliance with standard precautions than the traditionally-lab class.

## Methods

### Study design

This study used an experimental design.

### Study setting and sample

The study was conducted among nursing students at one of the largest universities in North Cyprus. The target comprised international students at the third and fourth- nursing levels because they have enough clinical practice credit hours and are close to graduation. The sample size was calculated using Raosoft sample calculator software, with a margin of error of 0.05, 95% confidence level, and 50% response distribution. The recommended minimum sample size was 100.

The total number of students at the third and fourth-nursing levels was 135, and 126 met the following inclusion criteria. They were to have attended and passed two main courses of the nursing program, Fundamentals of Nursing and Medical-Surgical Nursing, have a smartphone with an Android system, have Internet access, and have enough credit hours of clinical practice. Students who did not have the main professional courses or enough credit hours of clinical practice were excluded.

**Fig 1:**
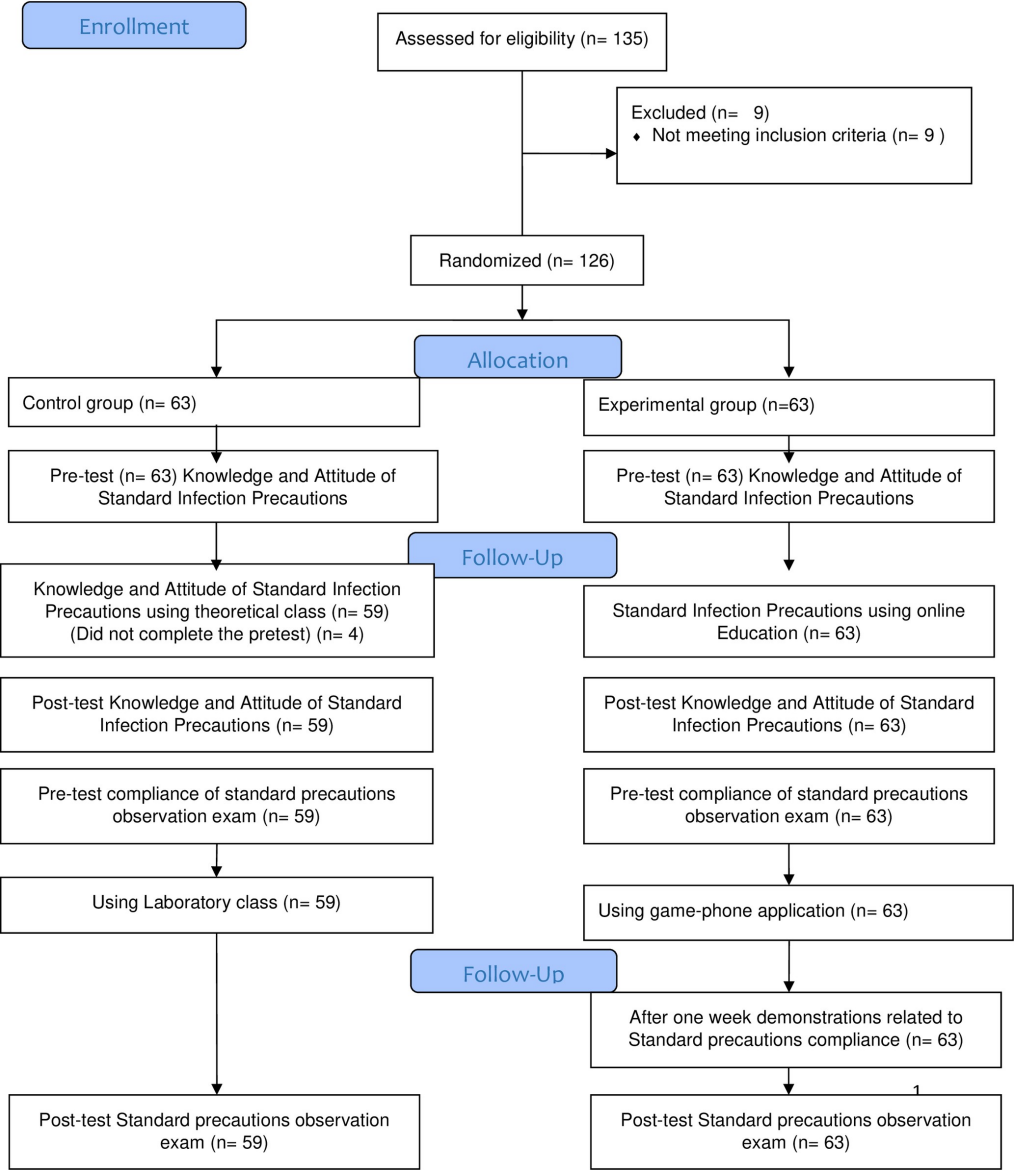
CONSORT flow diagram.

The students were divided randomly into the experimental and control groups. Each group began with 63 students; however, 4 students did complete the pre-test in the control group, and their data was dropped from the analysis. The experimental group’s participation rate was 100%, while the control group’s was 96%. See Fig 1.

### Study tool

Study researchers set up the tool package according to relevant literature and Centers for Disease Control and Prevention guidelines concerning knowledge, attitude, and compliance with standard precautions [1, 6–8].The demographic characteristics of the nursing students include age, gender, nursing level, and a previous infection control course. The knowledge of and attitudes toward standard precautions were developed based on previous studies [6, 8]; knowledge of standard precautions involved 18 items, and 16 items were attitudes toward standard precautions. The maximum high knowledge score was 18, “yes,” answers were recorded as 1, and “no” as 0. The cut-off points for good knowledge were > 9. The maximum high attitude score was 16, “yes,” answers were recorded as 1, and “no” received 0. The cut-off points for good attitude were > 8.

The last section included a checklist for compliance with standard precautions [7, 8]. The checklists involved 14 hand hygiene items, personal protective equipment, 11 items, and sharp safety, 10 items, and transmission-based precautions, 15 items. The observational checklist for compliance with standard precautions included 50 items with three options per item; completely done was 2, partially done was 1, and not done was 0. The total mean performance ranged from 0–100, with 50–100 categorizing high performance and 0–49 categorizing low performance. The cut-off point and pass level in practical exams were 50%.

A professor of infectious disease consultant examined all study tools to prevent inaccuracy and difficulty in understanding and validated them. The content validity analysis showed CVI scores above 0.87, demonstrating excellent content validity. The findings of the psychometrics revealed acceptable reliability and validity (Cronbach’s alpha = 0.74). The reliability consistency coefficients were 0.75, 0.73, 0.68, for knowledge, attitude, and compliance subscales, respectively.

The researchers prepared the educational content regarding standard precautions in line with the recent recommendations of the US Centers for Disease Control and Prevention 2016 [1]. Topics of the educational material included: emerging infectious diseases, hospital-acquired infections, infection control precaution guidelines, and standard precautions (hand hygiene, personal protective equipment, sharps safety, transmission-based precautions). Three experts in infection control reviewed the educational content to assess its content validity, and modifications were made based on the experts’ recommendations.

### Ethical approval

Ethical approval was obtained from the university’s institutional review board (No. 2019\71\877). Participants were given informed consent and received a code number to be more assured of privacy and complete confidentiality. Students were reassured that participation was voluntary and would not affect their grades. After the ethical approval, the study was carried out during the summer period of July and August 2019 to prevent clashing with midterm and final exam dates and lectures.

### Implementation

#### Experimental group

*Pre-Experiment*. The course began in July 2019. The study group received an email that included the link to create an account in the e-learning platform. A pre-test exam before the intervention was held in the classroom, with 40 minutes for completion, which included the first and second parts related to knowledge and attitude toward standard precautions. For the standard precautions observation exam, the principal investigator arranged the observations exam station in the fundamentals of nursing laboratory, which was included four stations. The first station assigned hand hygiene, the second station assigned personal protective equipment, the third station featured sharps safety, and the last station included transmission-based precautions. Each student approved the start of the observation exam to prevent forcing and stress. No feedback or correction was provided on their performance.

*Intervention*. Five live lessons lasting 10 to 15 minutes each were recorded and uploaded to the e-learning platform so students could re-listen and re-watch them. Quizzes followed each live lesson to ensure students had learned suitable knowledge and evaluated themselves. The total duration was 5 running weeks in July and August 2019.

The second intervention was related to standard precautions compliance using game-based virtual reality phone applications divided into two parts; the first part included a video regarding steps demonstration of standard precautions compliance checklists. The total duration of the video was 3 minutes. The second part was the game, created using Adobe Flash Professional CC and Adobe Flash CS6. The game had four scenarios; the first scenario was related hand hygiene (the 1min, 30s) followed by personal protective equipment (2 min, 15s), sharps safety (1min, 43s), and transmission-based precautions (3min, 23s). The total play game was 8 min, 11s.

To ensure that the game-based virtual reality phone application worked and downloaded correctly, the students downloaded game applications on their mobile phones in the fundamentals nursing laboratory according to the principal investigator’s instructions. After confirming that students downloaded the game correctly, they were given one week to play and re-play the games at will. During this week, the principal investigator randomly divided the students into 3 groups, with 21 students in each group, to enhance team-based learning. After completing the week, each group demonstrated the standard precautions compliance within two hours in the laboratory on the same day under the supervision of the principal investigator.

*Post-Intervention tests*. The students repeated the second part of the standard precautions’ knowledge and attitude test held in the classroom in August 2019. For the standard precautions observation exam, the student was asked to repeat the observation exam in a fundamentals nursing laboratory with the same evaluation criteria as the pre-test.

### Control group

*Pre-Intervention*. The course began in July 2019. The course was held for each group separately to prevent clashes between groups. The principal investigator arranged the classroom to perform the pre-test exam regarding knowledge and attitude of standard precautions. A standard precautions observation exam the same as for the experimental group was given. The pre-test exam items, observation exam, instructor, examiner, and completion were as same as the experimental group.

*Intervention*. Traditional teaching methods like PowerPoint presentation slides and printouts were applied for knowledge and attitude of standard precautions in the classroom. The students received five lectures; the duration of each lecture, the number of weeks, and the total lecture durations were the same as the experimental group. Each student received a hard copy of the material at the end of each topic. The students had a one-hour class in the laboratory, using PowerPoint presentation slides and hardcopy material to learn about complying with standard precautions. There were no further interventions or demonstrations related to standard precautions.

*Post-Intervention test*. The knowledge and attitude of standard precautions exams were repeated in the classroom, and a standard precautions observation exam was repeated the same as in the experimental group.

### Data analysis

Data were analyzed using the Statistical Package for Social Science (SPSS) version 20. Frequency and percentages were conducted to analyze the descriptive statistics of the participants. The Kolmogorov–Smirnov normality assessment test confirmed the normality distribution data, and parametric tests were used. An independent sample t-test was conducted for group comparison, and paired t-tests compare the score for each group before and after the intervention. Statistical significance was set at p< 0.05.

## Results

Table 1 presents the demographic statistics. The results illustrated that the mean age of students in the control group was 23.1± 8.3 years, while it was 22.9±6.8 years in the experimental group. Most participants were female in both groups. The fourth nursing class was featured, and most students in both groups had received previous infection control education. There was no statistically significant difference between both groups concerning descriptive characteristics at p > 0.05. Thus, the homogeneity was confirmed in both groups in the study.

**Table 1:** Descriptive characteristics of the control group and the experimental group.

Characteristics	Control Group (N═ 59)		Experimental Group (N═ 63)		P value
	N	%	N	%	
**Gender**					
Male	12	22.7	23	14.9	0.17a
Female	47	77.3	40	85.1	
**Nursing Class**					
Third Nursing Class	19	12.1	15	18.4	0.10 a
Fourth Nursing Class	40	87.9	48	81.6	
**Previous infection control education**					
Yes	43	88.2	51	92.4	0.19 a
No	16	11.8	13	7.6	
	Mean	SD	Mean	SD	
**Age**	23.1	8.3	22.9	6.8	0.34^b^

a: The chi-square; b: The independent-sample t test

An independent t-test of the mean scores of standard precautions knowledge, and attitude domains between the online education and traditional learning groups showed no statistically significant difference in mean scores between the two groups in the pre-test (p = 0.11, p = 0.13) respectively. However, after the intervention, the online education group had higher mean scores for standard precautions knowledge and attitude domains than the traditional lecture group (p = 0.001, p = 0.002).

In a pre-test and post-test comparison of the mean scores of standard precautions knowledge domain pretest (*M =* 9.5±2.4, posttest *M =* 12.3±1.1 p = 0.10), and attitude domain pretest (M = 6.1±1.4, posttest M = 9.3±2.5 p = 0.15) in the traditional learning group, the paired t-test showed no statistically significant improvement.

However, the online education group showed statistically significant improvement in mean scores of standard precautions knowledge domain pretest (M = 10.4±2.6, posttest M = 13.2±2.7 p = 0.002), and attitude domain (M = 8.7±2.2, posttest M = 11.2±1.5 p = 0.003) in the post-test compared with the pre-test (Table 2).

**Table 2:** Comparison of standard precautions knowledge and attitude means scores of the traditional lecture and the online education groups.

Standard Precautions Domains	Number of items	Groups	Pre-test	Post-test	P value **
			Mean Score ± SD	Mean Score ± SD	
Knowledge	18	Traditional group	9.5±2.4	12.3±1.1	0.10
		Online group	10.4±2.6	13.2±2.7	0.002
		P value *	0.11	0.002	
Attitude	16	Traditional group	6.1±1.4	9.3±2.5	0.15
		Online group	8.7±2.2	11.2±1.5	0.003
		P value *	0.013	0.001	
Overall	34	Traditional group	15.6±2.5	21.6±3.2	0.12
		Online group	19.1±1.7	24.4±2.5	0.001
		P value *	0.21	0.002	

*: The independent-sample t test

**: Paired t-test.

A comparison of the mean scores of standard precautions compliance domains between the game-based virtual reality phone application and laboratory class groups with the independent t-test showed no statistically significant difference in all compliance domains mean scores between the two groups in the pre-test (p = 0.21, p = 0.19, p = 0.11, p = 0.10 respectively). However, the results showed a statistically significant difference; the game-based virtual reality phone application group had higher mean scores of compliance domains than the traditional lecture group (p = 0.01, p = 0.01, p = 0.02, p = 0.04) (Table 3).

**Table 3:** Comparison of standard precautions compliance domains means scores of the laboratory class and the game-based virtual reality phone application.

Standard Precautions Compliance Domains	Number of items	Groups	Pre-observation	Post-observation	P value **
			Mean Score ± SD	Mean Score ± SD	
Hand Hygiene	14	Laboratory Class	5.4±1.6	9.3±2.3	0.23
		Game-Based Virtual Reality Phone Application	6.3±6.3	12.5 ±1.5	0.01
		**P value** *	0.21	0.01	
Personal Protective Equipment	11	Laboratory Class	6.2±4.1	8.6±1.2	0.11
		Game-Based Virtual Reality Phone Application	6.7±1.3	9.5±4.6	0.01
		**P value** *	0.19	0.01	
Sharps Safety	10	Laboratory Class	2.4±5.5	5.8±3.9	0.22
		Game-Based Virtual Reality Phone Application	4.2±4.7	7.8±7.7	0.04
		**P value** *	0.11	0.02	
Transmission-Based Precautions	15	Laboratory Class	6.1±3.8	7.9±6.6	0.19
		Game-Based Virtual Reality Phone Application	6.3±7.0	12.3±5.1	0.01
		**P value** *	0.10	0.04	
Overall	50	Laboratory Class	20.1±5.8	31.6±6.3	0.15
		Game-Based Virtual Reality Phone Application	23.5±7.9	42.1±5.6	0.02
		**P value** *	0.17	0.02	

*: The independent-sample t test

**: Paired t-test.

In the pre-test and post-test comparison of the mean scores of standard precautions in compliance domains in the traditional learning group, the paired t-test showed no statistically significant improvement. However, in the game-based virtual reality phone application, the results showed statistically significant improvement in mean scores of all compliance domains in the post-test compared with the pre-test (Table 3).

## Discussion

In this experimental design performed to assess the effectiveness of online instruction and game-based virtual reality phone application and traditional education on knowledge, attitude, and compliance with standard precautions among nursing students. The results showed significant improvement in both educational approaches the online education and game-based virtual reality phone applications compared with traditional education and traditionally-lab class.

The online education group showed significant compared with traditional education group in terms of knowledge and attitudes of standard precautions. The results confirmed the effectiveness of online teaching methods for nursing students in standard precautions education. In recent studies, the online teaching methods significantly improved nursing students related standard precautions knowledge and attitudes [18, 19, 36]. The significant improvement can be attributed to multiple topics and various teaching used for the intervention group. Moreover, online instruction provides opportunities for learners to repeat materials and use their knowledge in practice. Online learning focuses on learner-to-learner and teacher-to-student interaction so that students can obtain effective feedback at the right time [37, 38]. As online learning environments are improved with different technology ways, students expect teachers to keep technical proficiency in order to maintain online courses free of challenges such as technical issues and Interaction and engagement during using communication platforms [39].

Another finding was that the mean scores regarding compliance with standard precautions among participants who used game-based virtual reality phone applications were significantly increased compared to the traditionally-lab class. The significant improvements may be explained that the kind of these applications lets students re-watch and re-play at any place and time, allows students to learn to apply the standard precautions without errors according to the followed procedural steps so that it might have helped the student use this skill correctly during the standard precautions’ observation exam, and become more independent learning, where nursing students can go at their own pace, without the pressure of traditionally scheduled instruction [40, 41]. Studies in the relevant literature show the effectiveness of game-based virtual reality phone applications in education [25, 42]. In Bayram and Caliskan’s study, the experimental group improved more than the control group after using a game-based virtual reality phone application [25]. A study on nursing students showed that the game-based virtual reality application significantly improved advanced life support [42]. Tsai et al. assessed the effectiveness of computer games for increasing nurses’ understanding of chronic obstructive pulmonary disease, finding that their COPD care knowledge scores significantly improved after playing the game [27]. However, game-based virtual reality applications have proven valuable in teaching psychomotor skills and enhance the visual clinical environment, such as personal protective equipment and sharps safety, which are too tough to understand through a single observation in the traditionally-lab class. In addition the game virtual simulation offered an identical experience of a real clinical condition where they could see the effect of their clinical practice decisions in the virtual scenario without creating patient harm [43].

Conversely, the overall mean scores of the traditional group showed no statistically significant difference, which did not align with an experimental study performed among Jordanian nursing students in terms of infection prevention [36]. Traditional teaching is seen as monotonous and focuses on assessment paradigms that promote educators’ ability to repeat facts without really understanding the content. Educators may feel more comfortable using traditional teaching [29, 44]. However, educational methods should be improved in parallel with new technology to increase the effectiveness of nursing education, and encourage effective learning and improve students’ professional knowledge and skills.

The current study has limitations that need to be addressed. First, experiments produce artificial situations and results; results may only apply to one situation and may be difficult to replicate. Second, the study was implemented in one nursing school, and the results cannot be generalized. Third, no observations confirmed that students played the game entirely after leaving the laboratory.

## Conclusion

The results showed that integrated online education and game-based virtual reality phone applications showed statistically significant improvements toward standard precautions to traditional lectures. Based on the study results, using online education and game-based virtual reality phone application effectively transmits theoretical knowledge to practice among students and prepares them for professional life. This study contributes to a better understanding of the effectiveness of innovative educational methods in nursing education. The study results may help develop educational strategies in nursing. School managers and faculty members should improve methods used in nursing education in parallel with new technology to improve nursing students’ knowledge, attitude, and compliance regarding standard precautions. Qualitative research can be conducted to evaluate students’ views on new educational approaches more deeply in the future.

## Supporting information

S1 File (RAR)
